# Branched poly (trimethylphosphonium ethylacrylate‐*co*‐PEGA) by RAFT: alternative to cationic polyammoniums for nucleic acid complexation

**DOI:** 10.1002/jin2.50

**Published:** 2018-12-21

**Authors:** Alexander B. Cook, Raoul Peltier, Tammie R. Barlow, Joji Tanaka, James A. Burns, Sébastien Perrier

**Affiliations:** ^1^ Department of Chemistry University of Warwick Coventry CV4 7AL UK; ^2^ Syngenta, Jealott's Hill International Research Centre, Bracknell Berkshire RG42 6EY UK; ^3^ Faculty of Pharmacy and Pharmaceutical Sciences Monash University 381 Royal Parade Parkville Victoria 3052 Australia; ^4^ Warwick Medical School University of Warwick Coventry CV4 7AL UK

**Keywords:** Nucleic acids, Non‐viral gene delivery, Polyplex, Polymer Chemistry, RAFT polymerisation, Polyphosphonium, Branched, polymers

## Abstract

Cationic and highly branched poly (trimethylphosphonium ethylacrylate‐*co*‐poly (ethylene glycol) acrylate) (p (TMPEA‐*co*‐PEGA)), and its ammonium equivalent, have been synthesised from post‐polymerisation modification of a poly (bromo ethylacrylate‐*co*‐poly (ethylene glycol) acrylate) (p (BEA‐*co*‐PEGA)) precursor polymer produced using reversible addition fragmentation chain transfer (RAFT) polymerisation. The cationic polymers were evaluated for their ability to complex nucleic acids, their i*n vitro* cytotoxicity and their GFP pDNA transfection efficiency. The results show RAFT copolymerisation of BEA and PEGA is a simple route to polyphosphoniums showing reduced cytotoxicities and higher transfection efficiencies than their polyammonium alternatives.

## Introduction

The development of polymeric materials for non‐viral gene delivery applications has become a prolific area of research over the last two decades.(Whitehead et al., 2009) These compounds facilitate the use of nucleic acids such as siRNA, mRNA, and plasmid DNA as therapeutics to treat a range of diseases with currently limited traditional small molecule therapeutic options.(Fire et al., 1998) Historically, non‐viral gene delivery polymers have been cationic polymers or lipids that can compact negatively charged nucleotides to form nanoparticle polyplexes, which can be uptaken by cells. Commonly utilised systems include polyethyleneimine (PEI), chitosan, polylysine (PLL), and poly (dimethylamino ethylmethacrylate) (pDMAEMA).(Cook et al., 2018; Kanasty et al., 2013; Yin et al., 2014) The vast majority of polymer based nucleic acid delivery systems rely on amine‐based cationic moieties to complex the phosphate groups of the desired polynucleotides. Although polyamines and polyammoniums show good ability to complex nucleotides, they also have significant cytotoxic effects arising either immediately from the free cationic amine polymer or as a delayed toxicity associated with the intracellular processing of polyplexes.(Godbey et al., 1999; Godbey et al., 2001; Lv et al., 2006; Wolfert et al., 1999)

A number of strategies have been used to circumvent or alter the cytotoxicity of polyamine non‐viral vectors, most of which include variation of the amine p*K*_a_ or introduction of PEG units.(Pack et al., 2005) Using a cationic heterocycle, for example pyridinium or imidazolium, was shown to result in a better dispersion of the positive charge over the ring and a decreased toxicity.(Ilies et al., 2003) However, the use of non‐nitrogen centred cations for nucleic acid complexation has yet to be thoroughly explored.(Rose et al., 2017) Phosphonium containing compounds are generally less toxic than their ammonium equivalents due to differences in ionic radius and charge distribution.(Rose et al., 2017; Stekar et al., 1995; Strasak et al., 2017) In addition, phosphonium based lipids have been shown to transfect cells with high efficacies and lower toxicity than ammonium analogues.(Guenin et al., 2000) Interestingly, the few polymeric phosphonium materials reported so far in the literature showed a similar trend in relation to toxicity.(Hemp et al., 2012; Hemp et al., 2012; Ornelas‐Megiatto et al., 2012; Rose et al., 2015) Yet, this was only studied in the case of linear polymers.

The phosphonium containing polymers investigated as delivery vectors to date, show good transfection capabilities with lower toxicities, however the monomers used are either styrene based or required multistep syntheses and purification. The group of Long,(Hemp, Allen, et al., 2012; Hemp, Smith, et al., 2012) conducted research involving phosphonium containing monomers for nucleic acid complexation using modified styrene monomers, which can be difficult to polymerise, often require high temperatures and high initiator concentrations. Fréchet and coworkers, (Ornelas‐Megiatto et al., 2012) and also Rose *et al*.,(Rose et al., 2015) synthesised phosphonium containing monomers with triethylene glycol spacers between the (meth) acrylate group and the phosphonium moieties. While these monomers showed excellent promise for gene delivery applications, they typically require multistep syntheses and purification. Poly (bromo ethylacrylate) (pBEA) has recently been shown to be an excellent reactive precursor material for post polymerisation modification with a variety of functionalities, including phosphonium moeities.(Barlow et al., 2016) The monomer is compatible with controlled radical polymerisation techniques such as Reversible Addition Fragmentation chain Transfer (RAFT) polymerisation, which enable great control of molecular weight as well as access to complex architectures.(Boyer et al., 2009; Cobo et al., 2015) In addition, RAFT polymerisation with divinyl comonomers allows simple access to branched architectures,(Liu et al., 2005a) which have been shown to be more efficient gene delivery vectors than linear polymers.(Ahmed & Narain, 2013; Anderson et al., 2005; Wightman et al., 2001) In this contribution, we report the facile synthesis of branched phosphonium containing polymers using RAFT and a post‐polymerisation modification strategy. The highly branched p (TMPEA‐*co*‐PEGA), and its ammonium equivalent, were investigated for their ability to complex DNA, toxicity of the polymers, and their potential to transfect cells *in vitro* with GFP plasmid DNA.

## Experimental

### Materials

Poly (ethylene glycol) methyl ether acrylate with average *M*_n_ = 480 (PEGA), di (ethylene glycol) diacrylate (DEGDA), 4,4′‐azobis(4‐cyanovaleric acid) (ACVA), trimethylamine solution (31–35 wt. % in ethanol 4.2 M), trimethylphosphine solution (1.0 M in THF), polyethylenimine branched (bPEI, Mw ~25,000 by LS, Mn ~10,000 by SEC), agarose, ethidium bromide solution (500 μg/mL in H_2_O), deoxyribonucleic acid (DNA, low molecular weight from salmon sperm), were all obtained from Sigma‐Aldrich. All other materials were purchased from Fisher Scientific. Green fluorescent protein (GFP) expressing plasmid DNA (pWPI) Addgene plasmid #12254. Bromoethyl acrylate was synthesised according to a previously published procedure.(Barlow et al., 2016) The chain transfer agent (CTA), 2‐(((Butylthio)‐carbonothioyl)thio) propanoic acid (PABTC) was prepared according to a previously reported procedure.(Ferguson et al., 2005) 50X Tris‐Acetate‐EDTA (TAE) buffer for gel electrophoresis was made up at concentration of 2.0 M Tris acetate (Sigma Aldrich) and 0.05 M EDTA (Sigma Aldrich) in deionised water, pH 8.2–8.4, and stored at room temperature. Agarose loading buffer for samples (colourless) was made up at 30% (vol/vol) glycerol (Sigma Aldrich) in deionised water, and stored at room temperature. 2,3‐Bis(2‐methoxy‐4‐nitro‐5‐sulfophenyl)‐2H‐tetrazolium‐5‐carboxanilide inner salt (XTT sodium salt), and Phenazine methosulfate (PMS) were obtained from Sigma‐Aldrich.

### Characterisation

Size Exclusion Chromatography (SEC) was performed in DMF, using an Agilent 390‐LC MDS instrument equipped with differential refractive index (DRI), viscometry, dual angle light scattering, and dual wavelength UV detectors. The system was equipped with 2 x PLgel Mixed D columns (300 x 7.5 mm) and a PLgel 5 μm guard column. The eluent was DMF with 5 mmol NH_4_BF_4_ additive, and samples were run at 1 mL/min at 50 °C. Analyte samples were filtered through a nylon membrane with 0.22 μm pore size before injection. Apparent molar mass values (*M*_n,SEC_ and *M*_w,SEC_) and dispersity (*Đ*) of synthesized polymers were determined by DRI detector and conventional calibration using Agilent SEC software. Poly (methyl methacrylate) (PMMA) standards (Agilent EasyVials) were used for calibration. The Kuhn‐Mark‐Houwink‐Sakurada parameter *α*, relating to polymer conformation in solution was determined from the gradient of the double logarithmic plot of intrinsic viscosity as a function of molecular weight, using the SEC viscometry detector and Agilent SEC software. Proton nuclear magnetic resonance spectra (^1^H NMR) were recorded on a Bruker Advance 400 or 300 spectrometer (400 MHz or 300 MHz) at 27 °C, with chemical shift values (δ) reported in ppm, and the residual proton signal of the solvent used as internal standard. Proton‐decoupled carbon nuclear magnetic resonance spectra (^13^C NMR) were recorded on a Bruker Advance 400 (100 MHz) at 27 °C in CDCl_3_, with chemical shift values (δ) reported in ppm, and the residual proton signal of the solvent used as internal standard (δC 77.16). Phosphorous ^31^P NMR spectroscopy was performed on a Bruker Advance 400 (162 MHz) in DMSO‐d6, at 27 °C, with chemical shift values (δ) reported in ppm. Fourier transform infrared spectra (FTIR) were recorded on a Bruker Alpha FTIR ATR. Elemental analyses for CHN were carried out on a CE440 CHN elemental analyser, and bromine was analysed using classical oxygen flask methods by Warwick Analytical Service.

### Polymer synthesis

For a typical polymerisation, with the conditions [BEA]: [PEGA]: [DEGDA]: [CTA]: [I] = 40: 10: 2.5: 1: 0.1, CTA (PABTC, 10.7 mg, 0.0447 mmol), BEA (0.319 g, 1.78 mmol), PEGA (0.214 g, 0.446 mmol), DEGDA (23.9 mg, 0.112 mmol), ACVA (1.25 mg, 0.00447 mmol), and dioxane (0.445 mL) were added to a vial deoxegenated by bubbling with nitrogen and left to stir in an oil bath at 70 °C. After a predetermined time, the solution was removed from the oil bath and the polymer precipitated in diethyl ether (x3), and dried under vacuum. *M*_w_ = 127,900 g/mol, *Ð* = 5.4 (DMF SEC, +NH_4_BF_4_ additive eluent, DRI detector with PMMA calibration). ^1^H NMR spectrum (400 MHz, DMSO‐*d*_*6*_, δ ppm): 4.34 (m, 2H, ‐C (O)O‐C**H**_**2**_‐CH_2_‐Br), 4.12 (m, 2H, ‐C (O)O‐C**H**_**2**_‐CH_2_‐O‐), 3.65 (m, 2H, ‐C (O)O‐CH_2_‐C**H**_**2**_‐Br), 3.52 (m, 32H, ‐C**H**_**2**_‐ (PEG)), 3.24 (s, 3H, −(CH_2_‐CH_2_‐O)‐C**H**_**3**_), 2.39–1.54 (m, 3H, backbone), 0.89 (t, 3H, ‐C**H**_**3**_ (CTA)). ^13^C NMR spectrum (100 MHz, CDCl_3_, δ ppm): 173.79 (‐**C** (O)O‐), 70.26 (‐**C**H_2_‐ (PEG)), 66.82 (‐C (O)O‐**C**H_2_‐), 30.82 (‐**C**H_2_‐Br), 41.23 (backbone tertiary), 21.87 (backbone ‐**C**H2‐). FTIR *ν*cm^−1^: 2867 (medium, C‐H alkane), 1728 (strong, C=O ester), 1447 (medium, C‐H alkane), 1093 (strong, C‐O‐C PEG). 569 (weak, C‐Br). Elemental analysis shown in Table S2.

In order to further characterise the branched nature of the synthesised polymers, two linear p (BEA‐co‐PEGA) polymers were synthesised to give a comparison for the KMHS intrinsic viscosity vs molecular weight plots, and associated *α*values. The polymerisation conditions were identical to the above branched polymers, but without DEGDA crosslinker (conditions and characterisation data can be seen in the supporting information). *M*_n_ = 9,700 g/mol, *Ð* = 1.12; and *M*_n_ = 16,800 g/mol, *Ð* = 1.32; (DMF SEC, +NH_4_BF_4_ additive eluent, DRI detector with PMMA calibration). ^1^H NMR spectrum (400 MHz, DMSO‐*d*_*6*_, δ ppm): 4.34 (m, 2H, ‐C (O)O‐C**H**_**2**_‐CH_2_‐Br), 4.12 (m, 2H, ‐C (O)O‐C**H**_**2**_‐CH_2_‐O‐), 3.65 (m, 2H, ‐C (O)O‐CH_2_‐C**H**_**2**_‐Br), 3.52 (m, 32H, ‐C**H**_**2**_‐ (PEG)), 3.24 (s, 3H, −(CH_2_‐CH_2_‐O)‐C**H**_**3**_), 2.39–1.54 (m, 3H, backbone), 0.89 (t, 3H, ‐C**H**_**3**_ (CTA)). ^13^C NMR spectrum (100 MHz, CDCl_3_, δ ppm): 174.90 (‐**C** (O)O‐), 70.19 (‐**C**H_2_‐ (PEG)), 65.52 (‐C (O)O‐**C**H_2_‐), 31.32 (‐**C**H_2_‐Br), 41.28 (backbone tertiary), 22.87 (backbone ‐**C**H2‐). FTIR *ν*cm^−1^: 2880 (medium, C‐H alkane), 1725 (strong, C=O ester), 1440 (medium, C‐H alkane), 1095 (strong, C‐O‐C PEG), 576 (weak, C‐Br).

### Post‐polymerisation modification

Typical post‐polymerization substitution of branched p (BEA‐co‐PEGA) with trimethylamine: p (BEA‐co‐PEGA) (0.10 g of polymer, 0.447 mmol of BEA units) was dissolved in 2 mL of DMSO in a small vial with a stirrer bar, to which was added 2.5 equivalents of trimethylamine (4.2 M in ethanol, 266 μL, 1.12 mmol) and stirred for 48 h under a nitrogen atmosphere. Upon completion, the solution was concentrated by nitrogen flow, purified by precipitation into THF, and dried under vacuum, to give the desired p (TMAEA‐co‐PEGA). ^1^H NMR (400 MHz, DMSO‐*d*_*6*_, ppm): δ = 4.53 (m, 2H, ‐C (O)O‐C**H**_**2**_‐CH_2_‐NMe_3_), 4.12 (m, 2H, ‐C (O)O‐C**H**_**2**_‐CH_2_‐O‐), 3.91 (m, 2H, ‐C (O)O‐CH_2_‐C**H**_**2**_‐NMe_3_), 3.52 (m, 32H, ‐C**H**_**2**_‐ (PEG)), 3.34 (m, 9H, CH_2_‐CH_2_‐N**Me**_**3**_), 3.24 (s, 3H, −(CH_2_‐CH_2_‐O)‐C**H**_**3**_), 2.41–1.51 (m, 3H, backbone), 0.89 (m, 3H, ‐C**H**_**3**_ (CTA)). ^13^C NMR spectrum (100 MHz, CDCl_3_, δ ppm): 174.04 (‐**C** (O)O‐), 70.22 (‐**C**H_2_‐ (PEG)), 64.00 (‐C (O)O‐CH_2_‐**C**H_2_‐), 58.53 (‐C (O)O‐**C**H_2_‐CH_2_‐), 53.42 (‐N (**C**H_3_)_3_), 40.85 (backbone tertiary), 27.02 (backbone ‐**C**H2‐). FTIR *ν*cm^−1^: 2871 (medium, C‐H alkane), 1728 (strong, C=O ester), 1477 (medium, C‐H alkane), 1248 (medium, C‐N, amine), 1092 (strong, C‐O‐C PEG). Elemental analysis shown in Table S2.

Typical post‐polymerization substitution of branched p (BEA‐co‐PEGA) with trimethylphosphine: p (BEA‐co‐PEGA) (0.10 g of polymer, 0.447 mmol of BEA units) was disolved in 2 mL of DMSO in a small vial with a stirrer bar, to which was added 2.5 equiv of trimethylphosphine (1 M in THF, 1.12 mL, 1.12 mmol) and stirred for 48 h under a nitrogen atmosphere. Upon completion, the solution was concentrated by nitrogen flow, purified by precipitation into THF, and dried under vacuum, to give the desired p (TMPEA‐co‐PEGA). ^1^H NMR (300 MHz, DMSO‐*d*_*6*_, ppm): δ = 4.36 (m, 2H, ‐C (O)O‐C**H**_**2**_‐CH_2_‐PMe_3_), 4.13 (m, 2H, ‐C (O)O‐C**H**_**2**_‐CH_2_‐O‐), 3.51 (m, 32H, ‐C**H**_**2**_‐ (PEG)), 3.24 (s, 3H, −(CH_2_‐CH_2_‐O)‐C**H**_**3**_), 2.83 (m, 2H, ‐C (O)O‐CH_2_‐C**H**_**2**_‐PMe_3_), 2.07 (m, 9H, CH_2_‐CH_2_‐P**Me**_**3**_), 2.46–1.44 (m, 3H, backbone), 1.06 (m, 3H, ‐C**H**_**3**_ (CTA)). ^13^C NMR spectrum (100 MHz, CDCl_3_, δ ppm): 174.13 (‐**C** (O)O‐), 70.23 (‐**C**H_2_‐ (PEG)), 60.64 (‐C (O)O‐CH_2_‐**C**H_2_‐), 54.87 (‐C (O)O‐**C**H_2_‐CH_2_‐), 40.55 (backbone tertiary), 26.82 (backbone ‐**C**H2‐), 5.30 (‐P (**C**H_3_)_3_). FTIR *ν*cm^−1^: 2897 (medium, C‐H alkane), 1728 (strong, C=O ester), 1420 (medium, C‐H alkane), 1087 (strong, C‐O‐C PEG), 971 (strong, P‐CH_3_). Elemental analysis shown in Table S2.

### DLS/Zetapotential

Dynamic light scattering measurements were carried of resulting polymers and polyplexes at various N/P ratios using a Malvern nanoZS zetasizer instrument (scattering angle of 173°, 10 mW He‐Ne laser). For polyplex formation: appropriate amount of polymer stock solution and DNA stock solution were mixed and made up to a total volume of 1 mL in DI water (final concentration of polymer was 1 mg/mL, in all solutions). The resulting solutions were vortexed incubated for 30 minutes at room temperature and were analysed at 25 °C. Each sample was run in triplicate and data was acquired using the software (Malvern Zetasizer) provided. Zeta potential measurements were carried out on the same DLS samples at various N/P ratios using the same instrument, and Malvern disposable folded capillary cell (DTS1070) cuvettes.

### Agarose gel electrophoresis

Agarose gels (1% *w*/*v*) were prepared with agarose and 1 × TAE buffer. The solution was cooled on the bench for 5 minutes and 100 μL of 0.5 μg/mL ethidium bromide solution was added. The mixture was poured into the casted agarose tray and a comb inserted. The gel was left to set for a minimum of 30 minutes at room temperature. The agarose gels were run in 1× TAE buffer. The final gel was visualized under UV illumination at 365 nm using a UVP benchtop UV transilluminator system. Polyplexes of DNA were prepared at various N/P ratios. DNA stock solution of 60 μg/mL was prepared in PBS, and polymer stock solution of 300 μg/mL. For polyplex formation: appropriate amount of polymer stock solution and DNA stock solution were mixed and made up to a total volume of 100 μL in PBS (final concentration of DNA was 0.030 μg/μL, in all solutions). Polyplexes were vortexed and incubated at room temperature for 30 minutes. Prior to loading, 30 μL of loading buffer was added to each sample and 20 μL of polyplexes were loaded into the agarose gel wells. Gel electrophoresis was performed at 100 V for 30 minutes.

### Cell culture

3 T3 mouse endothelial cells were cultured in High Glucose DMEM (Dulbecco's Modified Eagle Medium) supplemented with 10% bovine calf serum and 1% of 2 mM glutamine. HEK293T cells were cultured in DMEM medium supplemented with 10% fetal bovine serum, 1% of 2 mM glutamine and 1% penicillin/streptomycin. The cells were grown as adherent monolayers at 310 K under a 5% CO_2_ humidified atmosphere and passaged at approximately 70–80% confluence.

### Cytotoxicity assays

For cell viability evaluation, 3 T3 cells were seeded in a 96 well plate at a density of 1 × 10^4^ cells per well. After 16 hours, the culture medium was replaced by fresh media containing a series of dilution of the polymers (2, 0.8, 0.2, 0.08, 0.02 mg/mL), prepared from stock solutions in media. Following 24 hours incubation, the medium was removed and replaced with fresh medium. The cells were incubated with a freshly prepared solution of XTT (0.2 mg/mL^−1^) and N‐methyl dibenzopyrazine methyl sulfate (250 μM) in medium for 16 hours. Absorbance of the samples was finally measured using a plate reader at 450 nm and 650 nm. The data presented are representative of a minimum of two independent experiments where each sample was measured in triplicate. Errors reported correspond to the standard deviation of the mean. Two tailed t‐tests were performed assuming equal variance.

### Transfection

Polyplex samples were prepared prior to incubation with the cells, via mixing of plasmid DNA solution (final concentration_DNA_ = 100 μg/mL) with the appropriate amount of polymer predissolved in sterile water (N/P ratio = 20), and left to complex at room temperature for one hour. HEK293T cells were seeded in a 24 well plate at a density of 1 × 10^5^ cells per well. After 16 hours, the culture medium was replaced by Optimem® cell culture media (Thermo Fisher Scientific) without fetal bovine serum. After one hour, the media was replaced by fresh Optimem® media containing the polyplex solutions (final concentration_DNA_ = 10 μg/mL), the cells left to incubate for 4 hours under 5% CO_2_ humidified atmosphere, then the media replaced with fresh culture media containing fetal bovine serum. Following overnight incubation, cells were washed with PBS, trypsinised, centrifuged, re‐dispersed in ice‐cold PBS and filtered into FACS tubes for analysis. Intracellular fluorescence was quantified using a BD LSR II cytometer (BD Biosciences) at excitation 488 nm and emission 525 nm. The geometric mean fluorescence was used as the sample value. The data in presented are representative of two separate experiments where each sample was measured in duplicate (*n* = 4). All errors reported correspond to the standard deviation from the mean. Two tailed t‐tests were performed assuming equal variance.

## Results and Discussion

**Figure 1:**
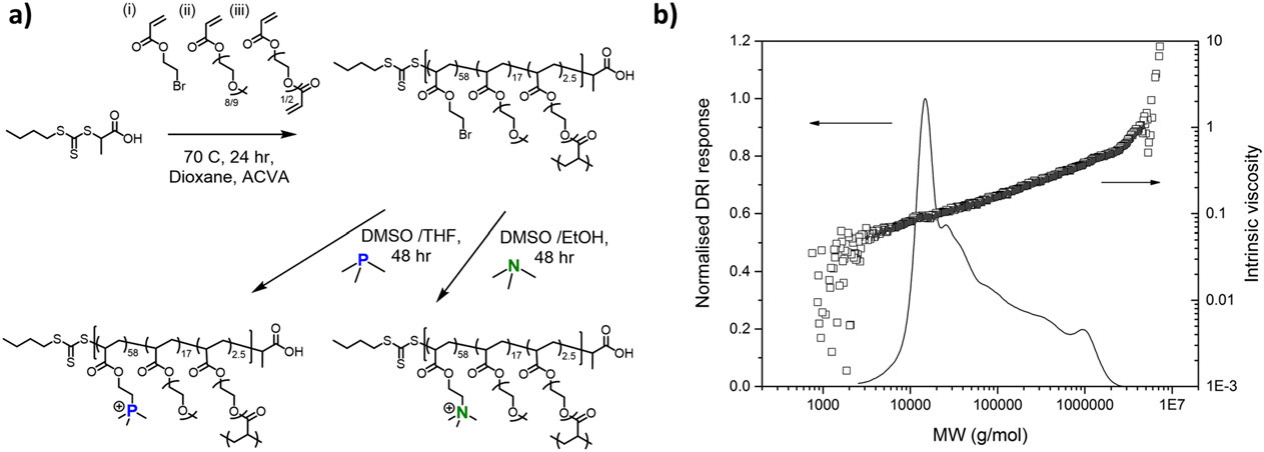
***a)** Reaction scheme for the RAFT polymerisation of (i) BEA and (ii) PEGA with (iii) crosslinker DEGDA, and subsequent post‐polymerisation modification with trimethylamine and trimethylphosphine; **b)** size exclusion chromatogram of p (BEA‐*co*‐PEGA) precursor polymer from refractive index detector, including KMHS plot of p (BEA‐*co*‐PEGA) from viscometry detector (DMF, PMMA calibration, Mn = 127,900 g/mol, Ð = 5.4, α = 0.35)*.

RAFT polymerisation was first used to synthesise highly branched structures by our group in 2005,(Liu et al., 2005a; Liu et al., 2005b; Semsarilar et al., 2010) following the considerable work of the Sherrington group,(Isaure et al., 2004; O'Brien et al., 2000; Slark et al., 2003) who developed facile and versatile branched polymeric systems using free radical polymerisation. Here, we employed the chain transfer agent, 2‐(((butylthio)carbonothioyl)thio) propanoic acid (PABTC) to copolymerise bromoethyl acrylate (BEA) and poly (ethylene glycol) acrylate (PEGA) with cross‐linker diethyleneglycol diacrylate (DEGDA) to form soluble highly branched polymers in a one‐pot methodology (Fig. 1a). A molar ratio of 80:20 BEA:PEGA was chosen, which achieves similar weight ratio (and therefore charge to PEG ratio) as previous reports in the literature,(Ornelas‐Megiatto et al., 2012; Rose et al., 2015) but from a facile copolymerisation strategy and post‐polymerisation modification. The RAFT polymerisation was conducted at 70 °C in dioxane, with the conditions [BEA]: [PEGA]: [DEGDA]: [CTA]: [I] = 40: 10: 2.5: 1: 0.1, and reached conversions of >90% in 24 hrs with no macroscopic gelation. Size exclusion chromatography (SEC) showed a broad molecular weight distribution and high molecular weights (*M*_w_ = 127,900 g/mol, *Ð* = 5.4) for the resulting polymers, as expected for highly branched polymeric systems (Fig. 1b). Information about the branched nature of these polymers and their globular conformation in solution can be obtained from viscometry detection on the SEC system. Figure 1b shows the Kuhn‐Mark‐Houwink‐Sakurada (KMHS) double Log plot of intrinsic viscosity against molecular weight. The gradient of the line (*α* value) gives information about the extent of polymer branching by inferring information about the polymer entanglements in solution. Linear polymers entangle more than branched polymers of similar molecular weight, leading to higher viscosity values which increase with increasing molecular weight more than equivalent branched systems. Linear polymers typically have *α* values of ~ 0.7. Here, linear p (BEA‐*co*‐PEG) was found to have *α* = 0.6–0.7 (**Fig. S2**) while the highly branched p (BEA‐*co*‐PEGA) reactive polymer precursor has an *α* = 0.35, indicating a highly branched polymer with a globular conformation.

**Figure 2:**
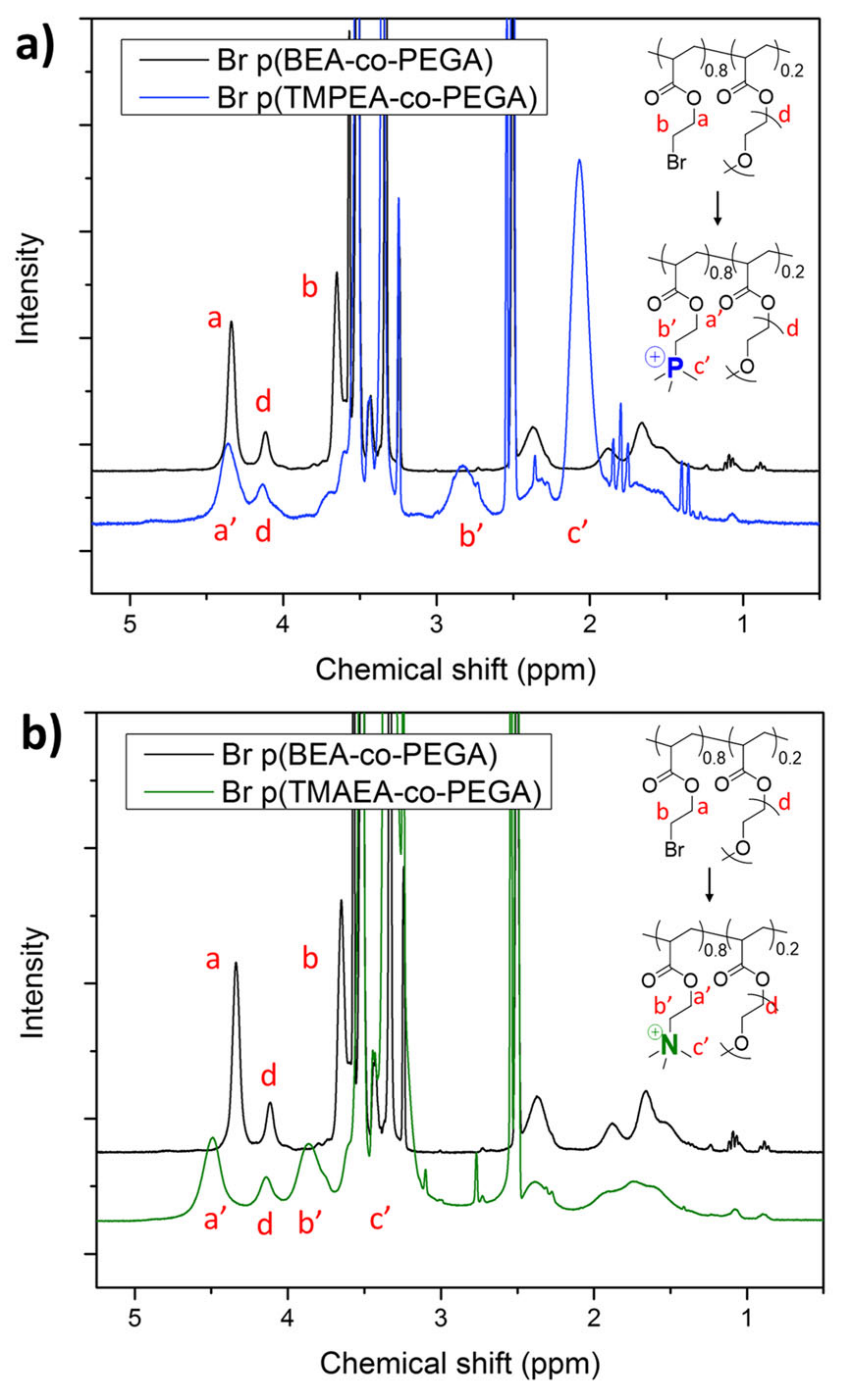
***a)*** ^*1*^ *H NMR spectra in DMSO‐d*_*6*_ *of branched p (BEA‐*co*‐PEGA) before and after substitution to form p (TMPEA‐*co*‐PEGA), **b)*** ^*1*^ *H NMR spectra in DMSO‐d*_*6*_ *of branched p (BEA‐*co*‐PEGA) before and after substitution to form p (TMAEA‐*co*‐PEGA)*.

With the highly branched reactive polymer precursor in hand, post‐polymerisation modification with trimethylamine and trimethylphosphine in DMSO/ethanol and DMSO/THF solvent mixtures, respectively, was used to form structurally equivalent polyphosphoniums and polyammoniums. Due to the highly charged nature of the resulting cationic polymers, purification from excess nucleophile could be achieved by precipitation of the charged polymer in THF. The lack of solubility in organic solvents of the resulting polymers, however, presented difficulties with characterisation of the molecular weight distribution using SEC. Using ^1^H NMR spectroscopy, substitution of the BEA alkyl halide with trimethylamine and trimethylphosphine nucleophiles was shown to proceed to high degrees of substitution of approximately >95% (Figs. 2, **S4** and **S6**), which agrees with previously reported results, however it was not possible to be more precise than this due to the broad nature of the peaks.(Barlow et al., 2016) Phosphorous (^31^P) NMR spectroscopy confirmed the presence of phosphonium moieties on the purified polymer (**Fig. S8**). In addition, elemental analysis was carried out to confirm the structure of both precursor BEA copolymer and post‐polymerisation modified polymers (**Table S2**). In the case of p (TMAEA‐*co*‐PEGA) appearance of nitrogen confirmed successful substitution. However, the technique was unable to provide a quantitative assessment of substitution value, as the generation of highly charged cationic moieties leads to the bromine anion being retained in the polymer as a counterion. Electrophoretic light scattering (zeta‐potential) was employed to determine the charge of branched p (BEA‐*co*‐PEGA) precursor, and substituted polymers. The charge increases from −3.05 mV for p (BEA‐*co*‐PEGA) to positively charged, p (TMAEA‐*co*‐PEGA) = +41.1 mV, p (TMPEA‐*co*‐PEGA) = +40.6 mV, further demonstrating substitution.

**Figure 3:**
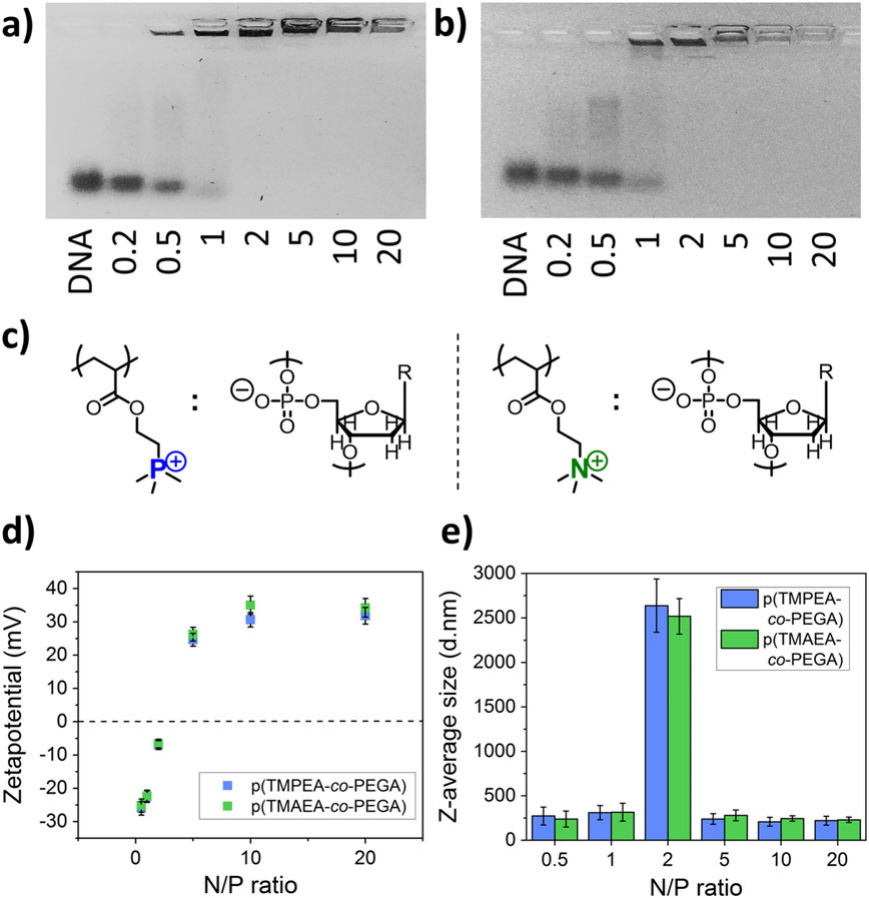
**a)** *p (TMPEA‐*co*‐PEGA) polyplex formation with DNA as characterised by agarose gel electrophoresis at varying P* ^*+*^ */P charge ratios; **b)** p (TMAEA‐*co*‐PEGA) polyplex formation with DNA as characterised by agarose gel electrophoresis at varying N* ^*+*^ */P charge ratios; **c)** structures of repeating units; **d)** surface charge (zetapotential) and **e)** size of polyplexes formed with DNA at varying N* ^*+*^ */P or P* ^*+*^ */P ratios as measured by dynamic light scattering and electrophoretic light scattering, respectively*.

Ability of the synthesised highly branched polymers to complex DNA was investigated using a combination of agarose gel electrophoresis, DLS, and zetapotential measurements. The formation of polyplexes at various N/P ratios, where N refers to nitrogens in the polymer/vector and P refers to negatively charged phosphate groups in the nucleic acid, was tested.(Felgner et al., 1997) For clarity, the different charge ratios are referred to as either P^+^/P for phosphonium polymers or N^+^/P quaternary ammonium polymers. **Figure** **3** **a** and **3** **b** show the agarose gel retardation assays for branched p (TMPEA‐*co*‐PEGA) and p (TMAEA‐*co*‐PEGA) respectively, with both polymers showing complexation of DNA at a charge ratio value of 2.

The overall size and surface charge of formed polyplexes was then determined using zetapotential and DLS measurements at various charge ratios (**Fig.** **3** **d** and **3** **e**). At low P^+^/P and N^+^/P ratios, observation of negatively charged polyplex nanoparticles with sizes of apporiximately 200 nm demonstrate the incomplete complexation of the oligonucleotide by both polymers, as excess of nucleic acid accounts for the negative charge observed. At a charge ratio of 2, both p (TMPEA‐*co*‐PEGA) and p (TMAEA‐*co*‐PEGA) form aggregates with sizes of over 2 μm with approximately neutral overall charges (~ ‐5 mV from zetapotential). At higher ratio values, branched polymers complex all the nucleic acid and form electrostatically stabilised charged polyplexes with sizes of around 200 nm and a surface charge of approximately +40 mV. Taken together, these results show that both p (TMPEA‐*co*‐PEGA) and p (TMAEA‐*co*‐PEGA) have very similar ability to complex nucleic acids.

**Figure 4:**
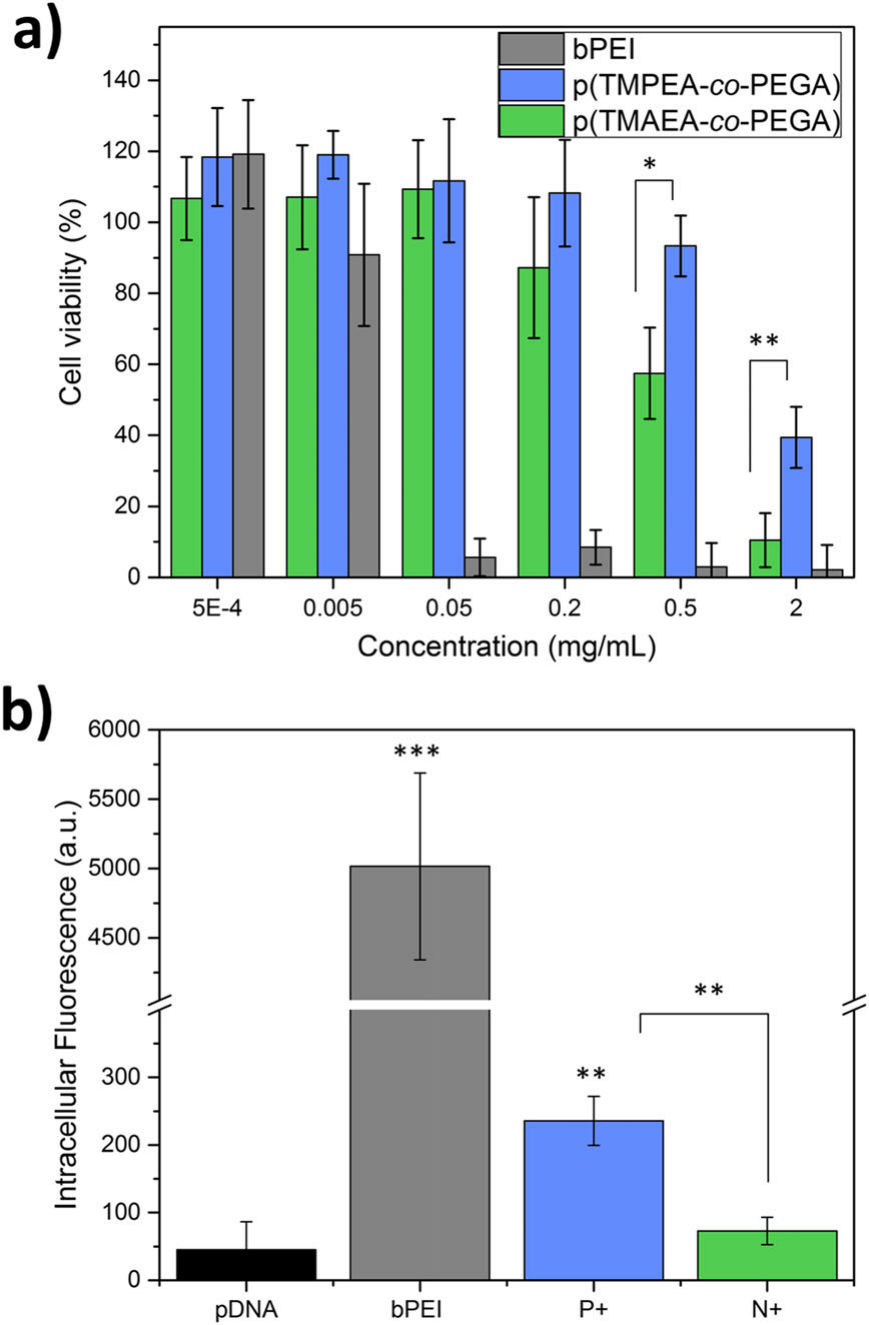
***a)** Cytotoxicity as determined by the percentage of cell viability for 3 T3 fibroblast cells treated with branched p (TMPEA‐*co*‐PEGA), branched p (TMAEA‐*co*‐PEGA), and bPEI for 24 hours at 37 °C. **b)** Transfection efficiency as determined by the intracellular fluorescence in HEK‐293 T cells following incubation with polyplexes of GFP‐plasmid DNA and branched p (TMPEA‐*co*‐PEGA), branched p (TMAEA‐*co‐PEGA) or commercial 25 k g/mol bPEI (N/P 20) for 4 hours at 37 °C and overnight incubation in polyplex‐free media, as measured by flow cytometry. *p < 0.05, **p < 0.01, ***p < 0.001.

Difference in the toxicity and transfection efficiency of phosphonium‐containing cationic polymers, as compared to ammonium equivalents, was investigated next. Acute toxicity of the polymers was assessed *in vitro* using 3 T3 fibroblast cell line as model. Cells were incubated with the polymers (0.5 μg/mL – 2 mg/mL) for 24 hr and viability was assessed using a typical protocol for the XTT assay (Fig. 4a). At concentrations of 5 μg/mL and below, all polymers present cell viability above 80%. While the control, commercial bPEI (25 k g/mol) shows significant toxicity at concentrations above 50 μg/mL, both p (TMPEA‐*co*‐PEGA) and p (TMAEA‐*co*‐PEGA) showed no adverse effects to cell viability at concentration as high as 0.5 and 0.2 mg/mL, respectively. The fact that phosphonium polymer is observed to be less toxic than the ammonium‐containing equivalent is in accordance with previous report in the literature. For example, Stekar *et al*. first reported that trimethylphosphonium cationic headgroups in phospholipids had lower cytotoxicity in mouse models compared to equivalent ammonium choline phospholipids, while the lipids also retained similar antineoplastic activity in both *in vitro* and *in vivo* induced carcinoma models.(Stekar et al., 1995) A similar increase in cell viability was observed for triethylphosphonium polymers as compared to triethylammonium polymers, for a range of polymer concentrations.(Ornelas‐Megiatto et al., 2012)

The polymers were subsequently assessed for their propensity to help deliver plasmid DNA encoding for green fluorescent protein (GFP) in cells. This was done by incubating model HEK293T cells with polyplexes, at a N/P ratio of 20, for 4 h, following which the cells were allowed to further grow for 16 hours prior to cellular fluorescence quantificatino using flow cytometry. The results, shown in Figure 4b, show that polyplexes of branched p (TMPEA‐*co*‐PEGA) enhance transfection efficiency by a factor of approximately six times as compared to naked pDNA. Interestingly, the transfection efficiency of branched p (TMAEA‐*co*‐PEGA) was shown to only increase the efficiency of naked pDNA by a factor of 3. Such a higher transfection efficiency for the phosphonium polymer when compared to the ammonium polymer follows the same trend as previously reported in the literature.(Ornelas‐Megiatto et al., 2012) Both of the synthesised branched polymers showed around 20‐fold lower transfection compared to commercial branched PEI. We hypothesise that this is due to the p (TMPEA‐*co*‐PEGA) and p (TMAEA‐*co*‐PEGA) polymers being too hydrophilic, and subsequently being unable to efficiently enter cells *via* endocytosis – a process which typically requires a balance of cationic charge and hydrophobicity.(Nelson et al., 2013) Hemp *et al*. reported a similar trend, where polymers with hydrophobic alkyl moieties attached to phosphonium cations had higher transfection efficiencies compared to more hydrophilic substituents.(Hemp, Allen, et al., 2012)

### Conclusions

In conclusion, we have demonstrated the facile synthesis of highly branched cationic poly (trimethylphosphonium ethylacrylate‐*co*‐PEGA) using a simple post‐polymerisation modification strategy of a poly (bromo ethylacrylate‐*co*‐PEGA) precursor polymer produced using RAFT polymerisation. This synthesis represents a significant improvement/simplification of current phosphonium containing polymer synthetic methods. The phosphonium and ammonium containing polymers were evaluated for ability to complex nucleic acids, polymer cytotoxicity, and pDNA transfection. The results show that phosphonium‐bearing polymers have increased biocompatibility and higher transfection efficiencies when compared to their exact ammonium equivalents. While transfection efficiencies were lower than those observed with commercial bPEI, their much lower toxicity highlights them as promising alternatives for gene delivery applications.

## Supporting information


**Table S1.** Polymerisation conditions for both branched and linear BEA PEGA copolymers, including characterisation data from SEC and ^1^H NMR spectroscopy.
**Figure S1.** Size exclusion chromatograms of branched and linear p (BEA‐co‐PEGA) precursor polymers using refractive index detection (DMF, PMMA calibration, Mn = 127,900 g/mol, Ð = 5.4).
**Figure S2.** KMHS plot of branched and linear p (BEA‐co‐PEGA) precursor polymers using viscometry detection in DMF.
**Figure S3.** ^13^C NMR spectrum of branched p (BEA‐co‐PEGA) precursor polymer in deuterated DMSO.
**Figure S4.** ^1^H NMR in DMSO‐d6 of branched p (TMPEA‐co‐PEGA).
**Figure S5.** ^13^C NMR in DMSO‐d6 of branched p (TMPEA‐co‐PEGA).
**Figure S6.** ^1^H NMR in DMSO‐d6 of branched p (TMAEA‐co‐PEGA).
**Figure S7.** ^13^C NMR in DMSO‐d6 of branched p (TMAEA‐co‐PEGA).
**Figure S8.** Phosphorous ^31^P‐NMR spectrum of branched p (TMPEA‐co‐PEGA) confirming the presence of phosphonium moieties on the purified polymer.
**Table S2.** Elemental analysis results for branched BEA PEGA copolymer, branched p (TMPEA‐co‐PEGA) and p (TMAEA‐co‐PEGA).
**Figure S9.** Proton ^1^H‐NMR spectra of branched p (TMPEA‐co‐PEGA) in D_2_O over 4 weeks at room temperature and pH 7, confirming no hydrolysis of polymer side chains occurring.
**Figure S10.** Representative DLS data for polyplex solutions (branched p (TMPEA‐co‐PEGA) with DNA, N/P 10, three repeats shown), **a)** intensity distribution, **b)** volume distribution, **c)** number distribution, **d)** correlograms, **e)** cumulants fit.
