# Supplementary material for: Branched poly (trimethylphosphonium ethylacrylate‐co‐PEGA) by RAFT: alternative to cationic polyammoniums for nucleic acid complexation
Source: J Interdiscip Nanomed. 2018 Dec 21;3(4):164–74. doi: 10.1002/jin2.50 (PMC6360508; doi:10.1002/jin2.50)
Supplement: Supplementary file 1 — Table S1. Polymerisation conditions for both branched and linear BEA PEGA copolymers, including characterisation data from SEC and 1H NMR spectroscopy. Figure S1. Size exclusion chromatograms of branched and linear p (BEA‐co‐PEGA) precursor polymers using refractive index detection (DMF, PMMA calibration, Mn = 127,900 g/mol, Ð = 5.4). Figure S2. KMHS plot of branched and linear p (BEA‐co‐PEGA) precursor polymers using viscometry detection in DMF. Figure S3. 13C NMR spectrum of branched p (BEA‐co‐PEGA) precursor polymer in deuterated DMSO. Figure S4. 1H NMR in DMSO‐d6 of branched p (TMPEA‐co‐PEGA). Figure S5. 13C NMR in DMSO‐d6 of branched p (TMPEA‐co‐PEGA). Figure S6. 1H NMR in DMSO‐d6 of branched p (TMAEA‐co‐PEGA). Figure S7. 13C NMR in DMSO‐d6 of branched p (TMAEA‐co‐PEGA). Figure S8. Phosphorous 31P‐NMR spectrum of branched p (TMPEA‐co‐PEGA) confirming the presence of phosphonium moieties on the purified polymer. Table S2. Elemental analysis results for branched BEA PEGA copolymer, branched p (TMPEA‐co‐PEGA) and p (TMAEA‐co‐PEGA). Figure S9. Proton 1H‐NMR spectra of branched p (TMPEA‐co‐PEGA) in D2O over 4 weeks at room temperature and pH 7, confirming no hydrolysis of polymer side chains occurring. Figure S10. Representative DLS data for polyplex solutions (branched p (TMPEA‐co‐PEGA) with DNA, N/P 10, three repeats shown), a) intensity distribution, b) volume distribution, c) number distribution, d) correlograms, e) cumulants fit. [file JIN2-3-164-s001.pdf]

Supporting Information for:

Branched poly(trimethylphosphonium ethylacrylate-*co*-PEGA) by RAFT: alternative to cationic polyammoniums for nucleic acid complexation

*Alexander B. Cook,[1] Raoul Peltier,[1] Tammie R. Barlow,[1] Joji Tanaka,[1] James A. Burns,[2] Sébastien Perrier[1,3,4]**

[1] Department of Chemistry, University of Warwick, Coventry, CV4 7AL, UK

[2] Syngenta, Jealott's Hill International Research Centre, Bracknell, Berkshire, RG42 6EY, UK

[3] Faculty of Pharmacy and Pharmaceutical Sciences, Monash University, 381 Royal Parade, Parkville, Victoria 3052, Australia

[4] Warwick Medical School, University of Warwick, Coventry, CV4 7AL, UK

*Corresponding author: Email: s.perrier@warwick.ac.uk; Tel: +44 2476 528 085

Table S1. *Polymerisation conditions for both branched and linear BEA PEGA copolymers, including characterisation data from SEC and ^{1}H NMR spectroscopy.*

**Polymer**	**Conditions**	**DRI SEC**			**TD SEC**		**^{1}H NMR**		
	**[BEA]:[PEGA]:[DEGDA]:[CTA]:[I]**	M_n **(g/mol)**	M_w **(g/mol)**	***Đ***	M_w **(g/mol)**	α	**DP** BEA	**DP** PEGA	M_n **(g/mol)**
Branched	40:10:2.5:1:0.1	23,600	127,900	5.41	220,900	0.35	58	17	-
Linear	64:16:0:1:0.1	9,700	10,800	1.12	13,900	0.6	39	9	11,300
Linear	240:60:0:1:0.1	16,800	22,100	1.32	31,800	0.78	160	35	45,400

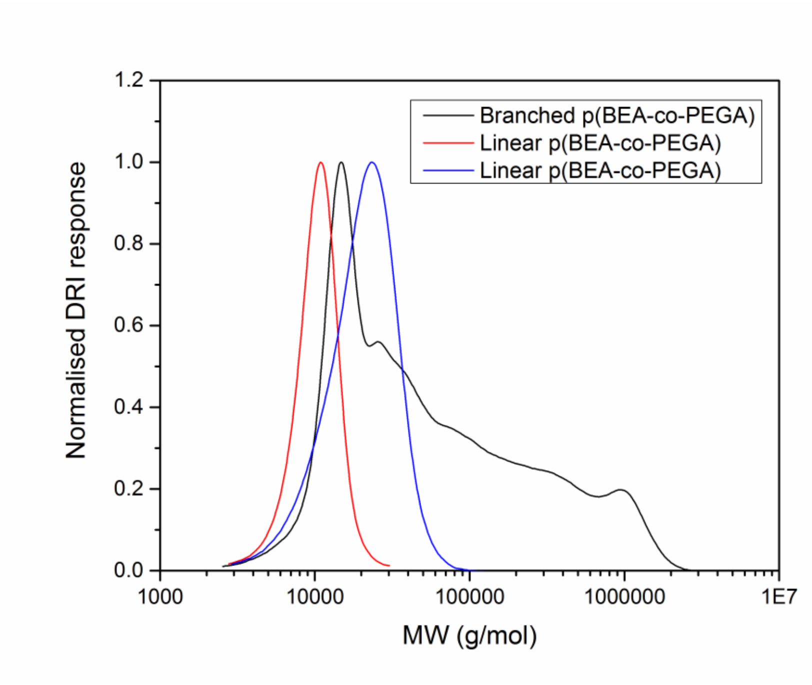

Figure S1. *Size exclusion chromatograms of branched and linear p(BEA-*co*-PEGA) precursor polymers using refractive index detection (DMF, PMMA calibration, Mn = 127,900 g/mol, Đ = 5.4).*

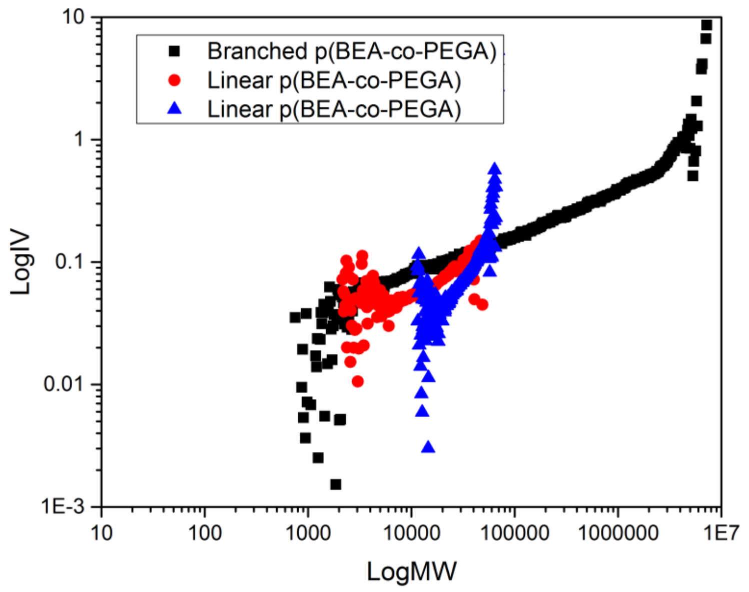

Figure S2. *KMHS plot of branched and linear p(BEA-*co*-PEGA) precursor polymers using viscometry detection in DMF.*

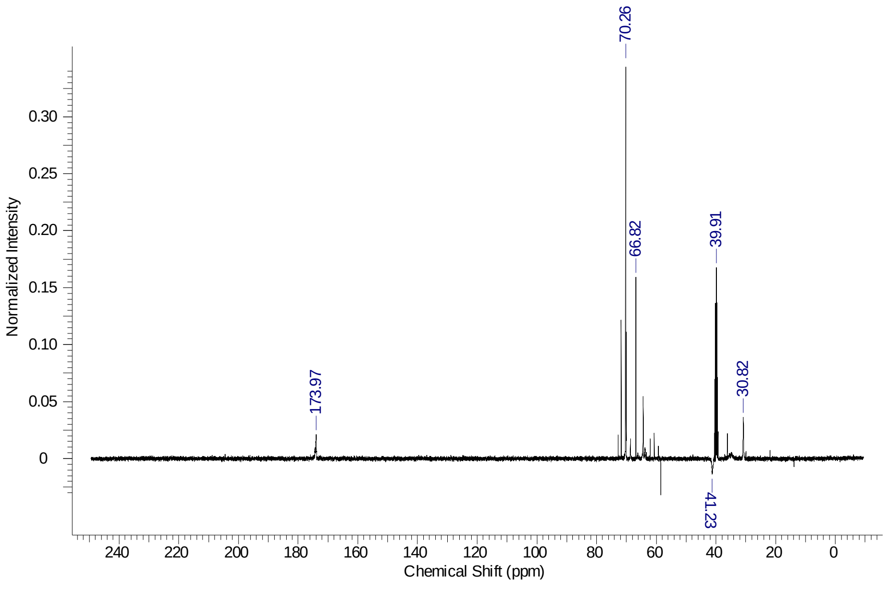

Figure S3. *^{13}C NMR spectrum of branched p(BEA-co-PEGA) precursor polymer in deuterated DMSO.*

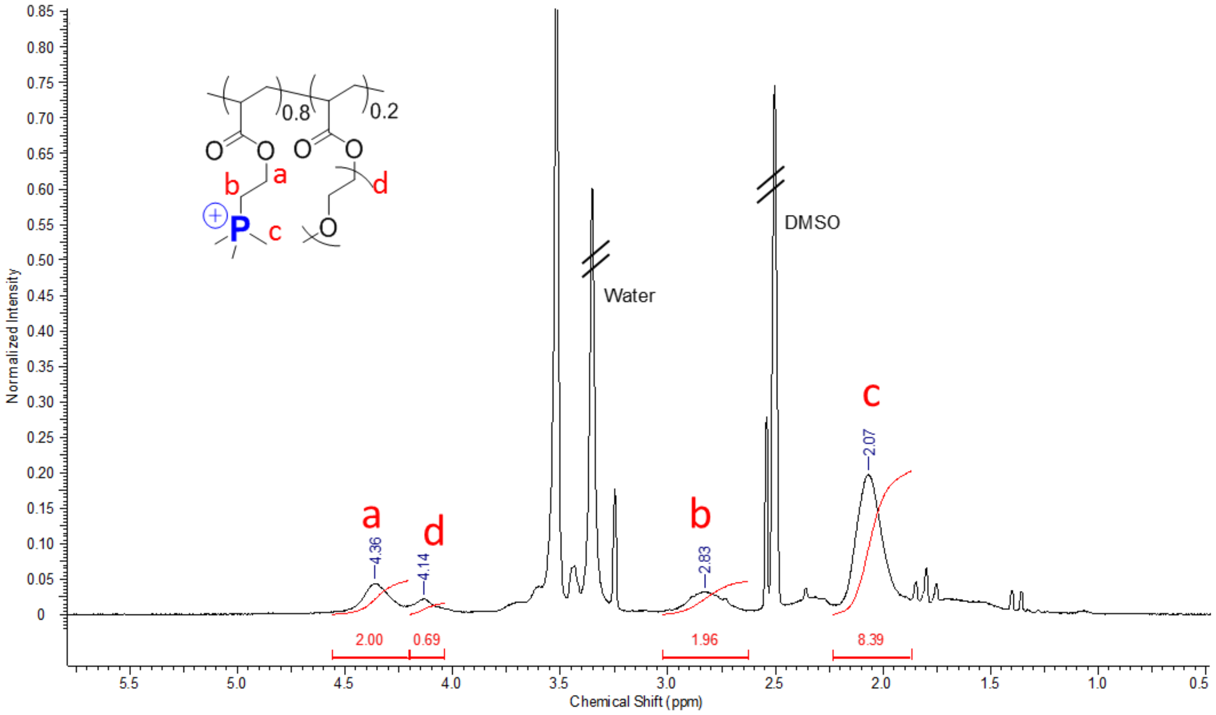

Figure S4. *^{1}H NMR in DMSO*-d6 *of branched p(TMPEA*-co-*PEGA).*

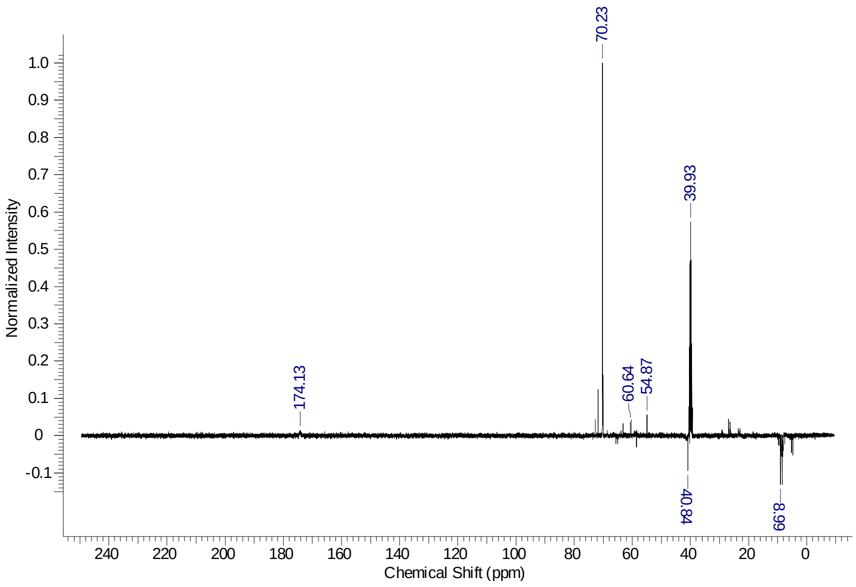

Figure S5. *^{13}C NMR in DMSO-d6 of branched p(TMPEA-co-PEGA).*

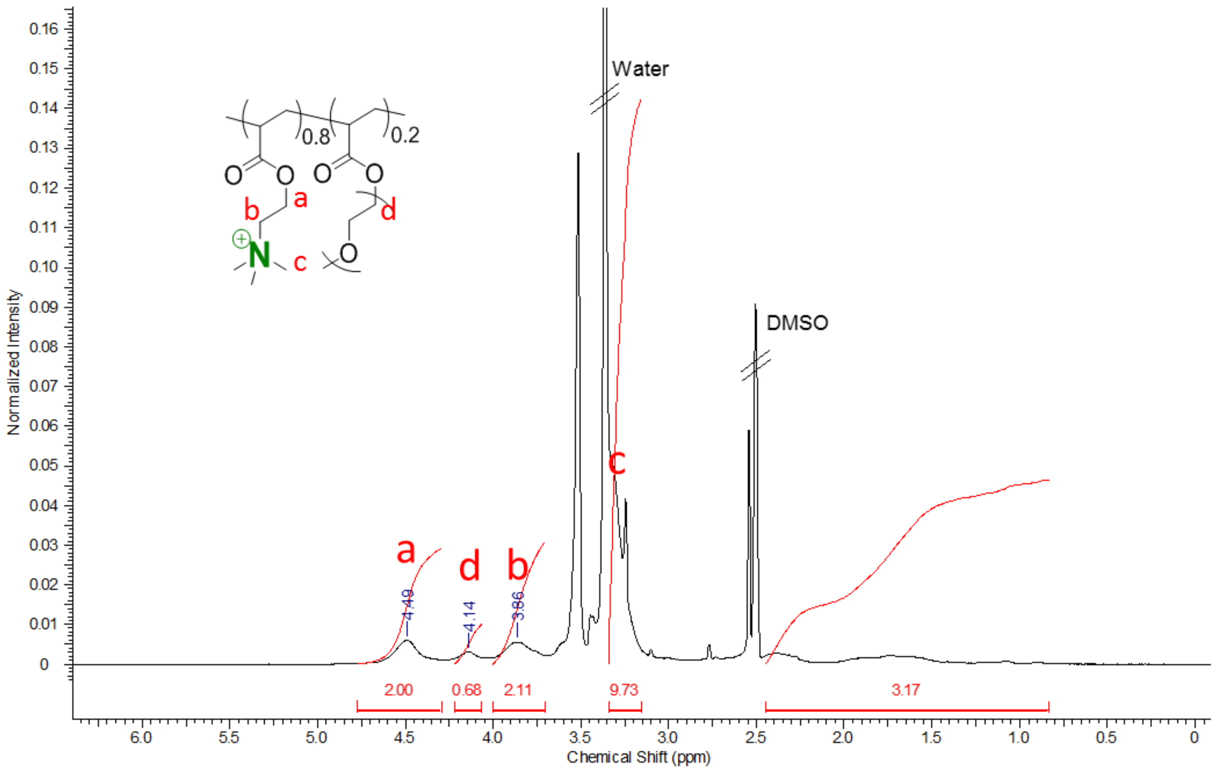

Figure S6. *^{1}H NMR in DMSO*-d6 *of branched p(TMAEA*-co-*PEGA).*

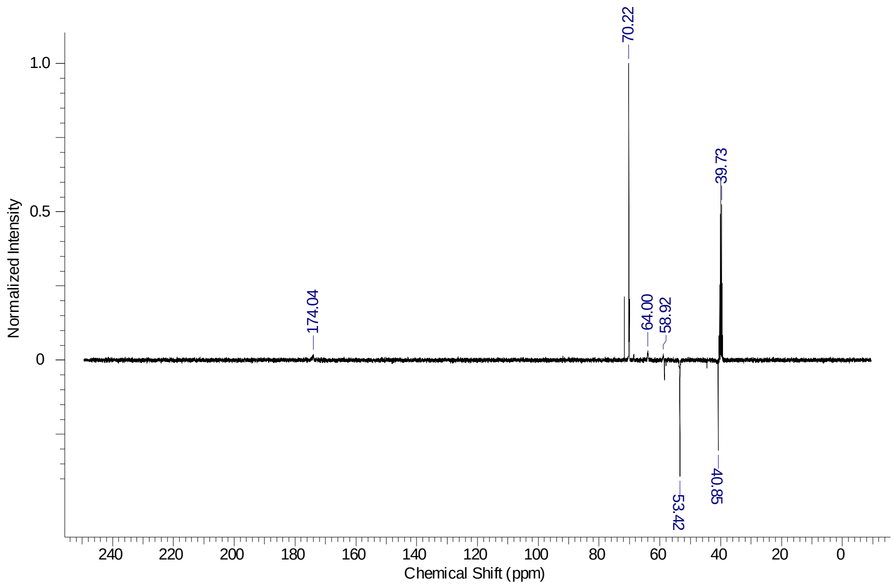

Figure S7. *^{13}C NMR in DMSO-d6 of branched p(TMAEA-co-PEGA).*

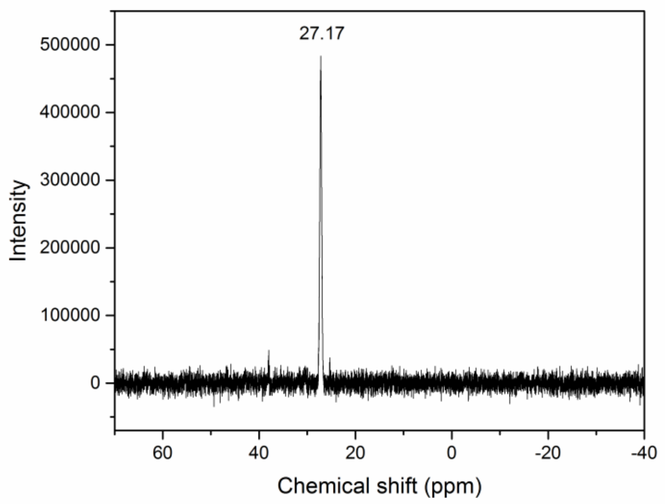

Figure S8. *Phosphorous ^{31}P-NMR spectrum of branched p(TMPEA*-co-*PEGA) confirming the presence of phosphonium moieties on the purified polymer.*

Table S2. *Elemental analysis results for branched BEA PEGA copolymer, branched p(TMPEA*-co-*PEGA) and p(TMAEA*-co-*PEGA).*

**Polymer**	**C (%)**		**H (%)**		**N (%)**		**Br (%)**		**S (%)**	
	Calc.	Found	Calc.	Found	Calc.	Found	Calc.	Found	Calc.	Found
**Branched p(BEA-co-PEGA)** $C_{445.5}H_{739}Br_{40}O_{198.25}S_3$	42.60	41.95	5.93	6.08	0.00	0.00	25.45	24.91	0.77	1.01
**Branched p(TMAEA-co-PEGA)** $C_{565.5}H_{1099}Br_{40}N_{40}O_{198.25}S_3$	45.51	40.45	7.42	7.79	3.75	3.10	21.42	19.88	0.64	1.99
**Branched p(TMPEA-co-PEGA)** $C_{565.5}H_{1099}Br_{40}O_{198.25}P_{40}S_3$	43.53	38.04	7.10	7.22	0.00	0.00	20.48	19.21	0.62	1.10

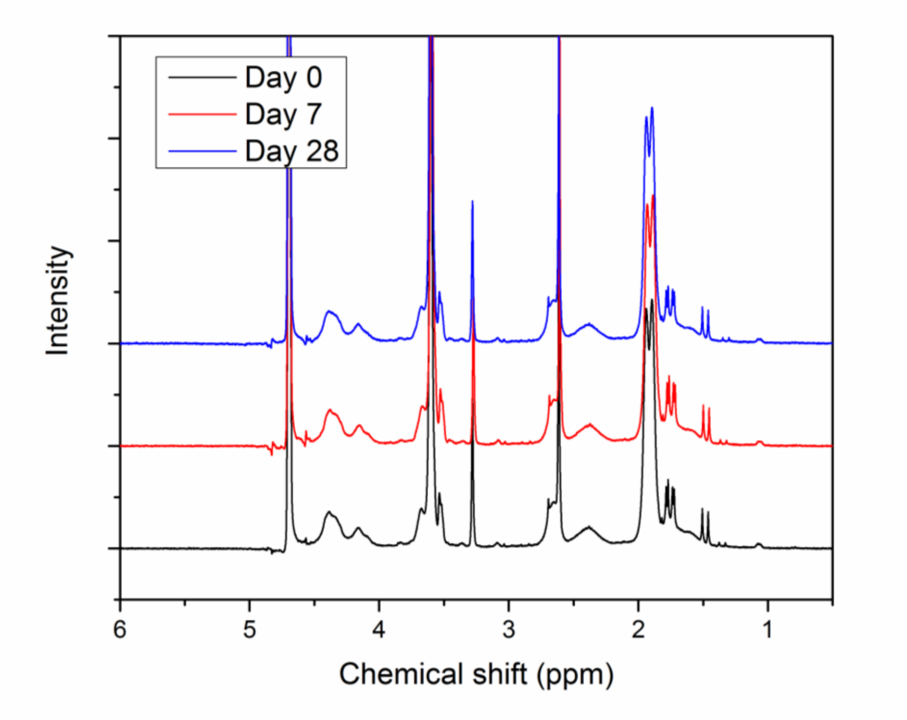

Figure S9. *Proton ^{1}H-NMR spectra of branched p(TMPEA*-co-*PEGA) in D_2O over 4 weeks at room temperature and pH 7, confirming no hydrolysis of polymer side chains occurring.*

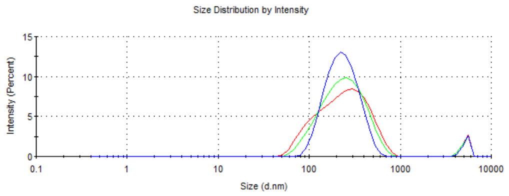

a)

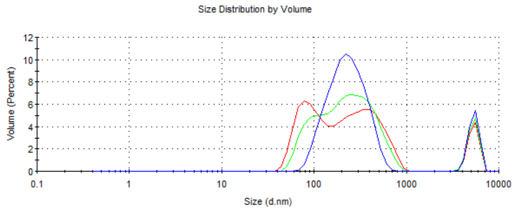

b)

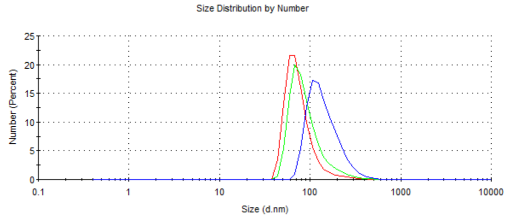

c)

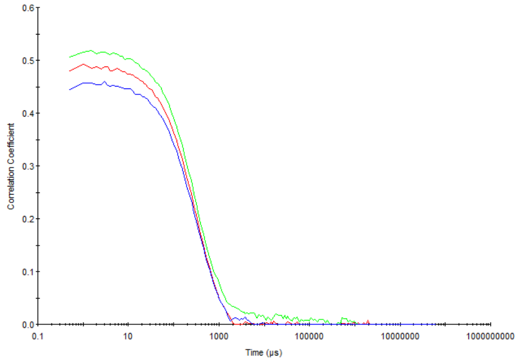

d)

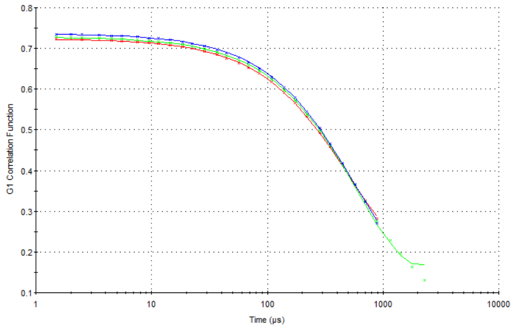

e)

Figure S10. Representative DLS data for polyplex solutions (branched p(TMPEA-co-PEGA) with DNA, N/P 10, three repeats shown), **a)** intensity distribution, **b)** volume distribution, **c)** number distribution, **d)** correlograms, **e)** cumulants fit.
